# Pharmacokinetic/pharmacodynamic issues for optimizing treatment with beta-lactams of Gram-negative infections in critically ill orthotopic liver transplant recipients: a comprehensive review

**DOI:** 10.3389/frabi.2024.1426753

**Published:** 2024-06-17

**Authors:** Milo Gatti, Federico Pea

**Affiliations:** ^1^ Department of Medical and Surgical Sciences, Alma Mater Studiorum University of Bologna, Bologna, Italy; ^2^ Clinical Pharmacology Unit, Department for Integrated Infectious Risk Management, Istituto di Ricovero e Cura a Carattere Scientifico (IRCCS) Azienda Ospedaliero-Universitaria di Bologna, Bologna, Italy

**Keywords:** orthotopic liver transplant, critically ill patients, antibiotic, beta-lactams, pharmacokinetic/pharmacodynamic optimization, TDM-guided approach

## Abstract

Orthotopic liver transplant (OLT) represents the standard of care for managing patients affected by end-stage and life-threatening liver diseases. Although a significant improvement in surgical techniques, immunosuppressant regimens, and prompt identification of early post-transplant complications resulted in better clinical outcome and survival in OLT recipients, the occurrence of early bacterial infections still represents a remarkable cause of morbidity and mortality. In this scenario, beta-lactams are the most frequent antimicrobials used in critical OLT recipients. The aim of this narrative review was to provide a comprehensive overview of the pathophysiological issues potentially affecting the pharmacokinetics of beta-lactams and to identify potential strategies for maximizing the likelihood of attaining adequate pharmacokinetic/pharmacodynamic (PK/PD) targets of beta-lactams in critically ill OLT recipients. A literature search was carried out on PubMed-MEDLINE database (until 31^st^ March 2024) in order to retrieve clinical trials, real-world observational evidence, and/or case series/reports evaluating the PK/PD of traditional and novel beta-lactams in settings potentially involving critically ill OLT recipients. Retrieved evidence were categorized according to the concepts of the so-called “antimicrobial therapy puzzle”, specifically assessing a) beta-lactam PK/PD features, with specific regard to aggressive PK/PD target attainment; b) site of infection, with specific regard to beta-lactam penetration in the lung, ascitic fluid, and bile; and c) pathophysiological alterations, focusing mainly on those specifically associated with OLT. Overall, several research gaps still exist in assessing the PK behavior of beta-lactams in critical OLT recipients. The impact of specific OLT-associated pathophysiological alterations on the attainment of optimal PK/PD targets may represent an important field in which further studies are warranted. Assessing the relationship between aggressive beta-lactam PK/PD target attainment and clinical outcome in critical OLT recipients will represent a major challenge in the next future.

## 1 Introduction

Orthotopic liver transplant (OLT) represents the standard of care for managing patients affected by end-stage and life-threatening liver diseases (Lucey et al., 2023). In the last years, consistent improvement in surgical OLT techniques, in immunosuppressant regimens, and in identifying promptly early post-transplant complications lead to a remarkable reduction of unfavorable outcomes (i.e., perioperative death, ischemia-reperfusion syndrome, primary allograft non-function) (Shafiekhani et al., 2019; Lucey et al., 2023). However, unfortunately early post-transplant bacterial infections still represent a major cause of morbidity and mortality (Patel and Huprikar, 2012; Shafiekhani et al., 2019; Lucey et al., 2023). Specifically, surgical complications affecting the vascular and/or the biliary anastomoses, multiorgan failure requiring prolonged mechanical ventilation and/or renal replacement therapy, and poor wound healing are considered major risk factors of bacterial infectious complications in the early post-transplant period (Patel and Huprikar, 2012; Fernandez and Gardiner, 2015; Lucey et al., 2023). The extension of the possibility of donating liver not only after declared brain death (DBD), bur also after circulatory death (DCD) added a further risk, as the prevalence of allograft-associated infectious complications after OLT was shown to be higher in this latter population (Tu et al., 2016; Zhang et al., 2016; Croome and Taner, 2020; Lucey et al., 2023).

Healthcare-associated bacterial infections represent the vast majority of early post-transplant infections in critically ill OLT recipients (Patel and Huprikar, 2012). Specifically, hospital-acquired pneumonia (HAP), ventilator-associated pneumonia (VAP), bloodstream infections (BSIs), surgical site infections, and biliary/intra-abdominal infections are those most frequently reported (Weiss et al., 2010; Ikegami et al., 2012; Karapanagiotou et al., 2012; Patel and Huprikar, 2012; Van Delden, 2014; Antunes et al., 2015; Laici et al., 2018; Chueiri Neto et al., 2019; Massa et al., 2019; Wu et al., 2022). The prevalence of the different causative pathogens may greatly vary among the different transplant centers worldwide (Patel and Huprikar, 2012), but that of Gram-negatives is worryingly increasing nowadays (Patel and Huprikar, 2012; Giannella et al., 2023; Taddei et al., 2023). Unfortunately, most of these may be multidrug-resistant (MDR) or difficult-to-treat resistant (DTR) pathogens, and this pattern of resistance may affect clinical outcome, and cause prolonged antibiotic treatment (Patel and Huprikar, 2012).

In the scenario of Gram-negative infections occurring in OLT recipients, both traditional and novel beta-lactams may currently represent first-line therapy (Aguado et al., 2018). Prompt dosing optimization may play a key role for maximizing the probability of pharmacokinetic/pharmacodynamic (PK/PD) target attainment of beta-lactams and for improving clinical outcome (Abdul-Aziz et al., 2020). Notably, in OLT recipients specific pathophysiological/iatrogenic alterations may add further complexity to that commonly depending on critically illness (Roberts et al., 2012; Blot et al., 2014; Roberts et al., 2014), namely bleeding requiring multiple transfusions, cytokine release syndrome associated with DCD transplant, need for continuous renal replacement therapy [CRRT] with oXiris filter for reducing bilirubin plasma levels. All of these alterations may significantly affect the pharmacokinetic behavior of beta-lactams, so that implementing a therapeutic drug monitoring (TDM)-guided expert clinical pharmacological advice (ECPA) program may be helpful for personalizing therapy with beta-lactams and for optimizing PK/PD target attainment in each single critically ill OLT recipient having Gram-negative infections (Gatti and Pea, 2023b).

The aim of this narrative review was to provide a comprehensive overview of the pathophysiological issues potentially affecting the pharmacokinetics of beta-lactams and to identify potential strategies for maximizing the likelihood of attaining adequate PK/PD targets of beta-lactams in critically ill OLT recipients.

## 2 Methods

A literature search was carried out on PubMed-MEDLINE database (until 31^st^ March 2024) in order to retrieve clinical trials, real-world observational evidence, and/or case series/reports evaluating the PK/PD of traditional and novel beta-lactams in settings potentially involving critically ill OLT recipients. The following terms were searched alone and/or in combination on PubMed-MEDLINE: “orthotopic liver transplant; OLT recipients; critically ill OLT recipients; beta-lactams; piperacillin-tazobactam; meropenem; ceftazidime; cefepime; ceftazidime-avibactam; ceftolozane-tazobactam; meropenem-vaborbactam; imipenem-relebactam; cefiderocol; blood loss; blood transfusion; continuous renal replacement therapy, hemofilter adsorption; hemofiltration adsorption; oXiris; Cytosorb; cytokine release syndrome; donation after circulatory death; DCD; lung penetration; ELF penetration; epithelial lining fluid; biliary penetration; abdominal penetration; peritoneal fluid penetration; pharmacokinetic/pharmacodynamic; PK/PD; therapeutic drug monitoring; TDM”. No language or time restrictions were applied.

## 3 Optimizing PK/PD target attainment of beta-lactams in critically ill OLT recipients according to the so-called “antimicrobial therapy puzzle” concepts

Optimizing therapy with beta-lactams in critically ill OLT recipients strictly requires to apply the so-called “antimicrobial therapy puzzle” concepts (Pea and Viale, 2006), in which different components should merge, namely the PK/PD features of antibiotics, the site of infection, the clinical isolate with its susceptibility to the antibiotic in terms of minimum inhibitory concentration (MIC) value, and the pathophysiological alterations of the patient.

To this regard, it is noteworthy to highlight that in critically ill OLT recipients some specific features are most likely reported compared with general intensive care unit (ICU) patients. Specifically, intra-abdominal and/or biliary infections are prevalent among critically ill OLT recipients, and may account for up to 50% of early infections occurring after OLT (Van Delden, 2014; Taddei et al., 2023).

**Figure 1:**
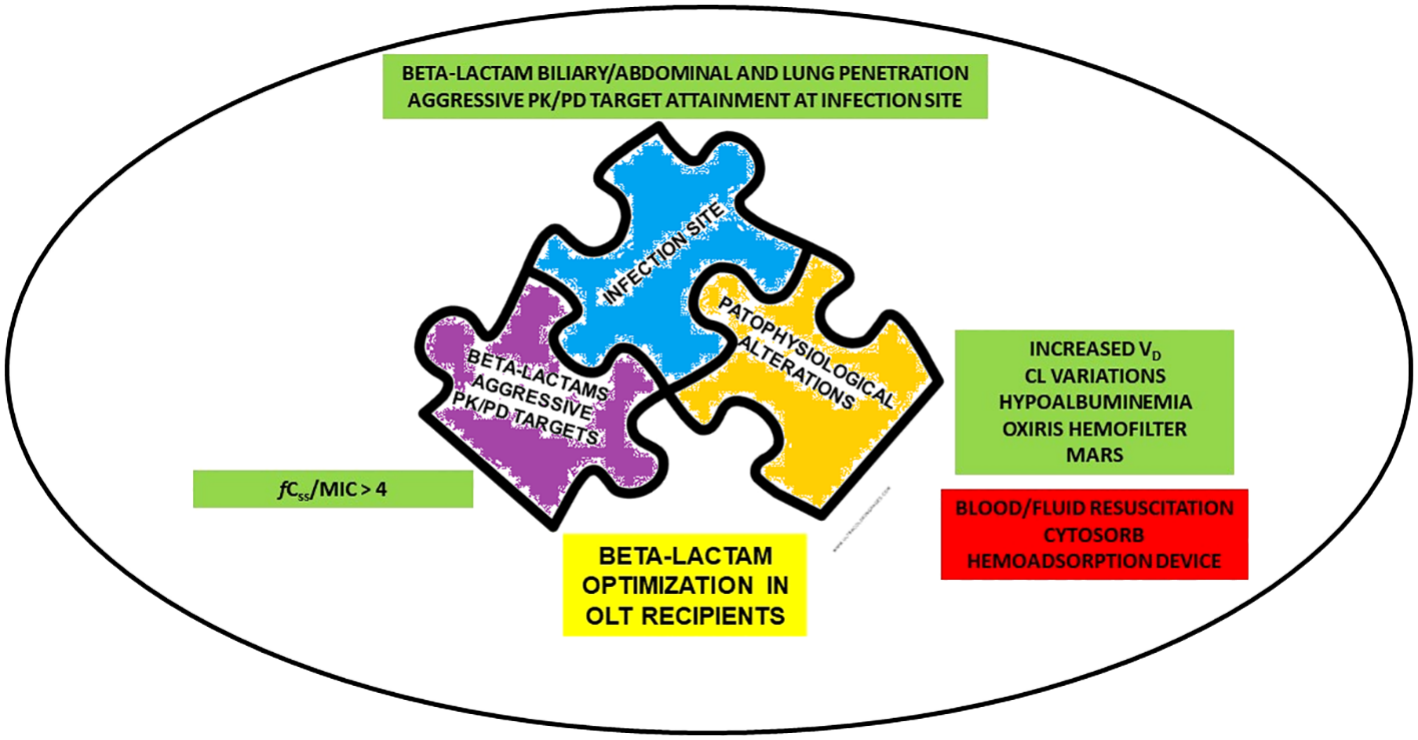
Determinants for beta-lactams optimization in critical OLT recipients according to “antimicrobial therapy puzzle” concepts. CL, clearance; *f*C_ss_, free steady-state concentrations; MARS, Molecular Adsorbent Recirculating System; MIC, minimum inhibitory concentration; OLT, orthotopic liver transplant; PK/PD, pharmacokinetic/pharmacodynamic; V_D_, volume of distribution.

Retrieved evidence were categorized according to the concepts of the so-called “antimicrobial therapy puzzle” (Pea and Viale, 2006) namely: a) beta-lactam PK/PD features, with specific regard to aggressive PK/PD target attainment; b) site of infection, with specific regard to beta-lactam penetration in the lung, ascitic fluid, and bile; c) pathophysiological alterations, focusing mainly on those specifically associated with OLT. Notably, the “antimicrobial therapy puzzle” concepts may be entirely translated in the challenging scenario of critical OLT recipients, as detailed in **Figure 1**.

### 3.1 Optimal PK/PD target attainment for beta-lactams

Beta-lactams exhibit time-dependent antibacterial activity, and the percentage of time of the dosing interval in which the free (unbound) concentration remains above the minimal inhibitory concentration (MIC) (%*f*T_>MIC_) is considered as the best PK/PD index predicting bacterial killing (Tilanus and Drusano, 2023).

According to preclinical and clinical evidence, a paradigm shift is currently happening in the concept of PK/PD optimization of beta-lactams (Gatti and Pea, 2023a). Generally, conservative targets of 40–100%*f*T_>MIC_ were commonly adopted in pivotal trials for granting clinical efficacy with beta-lactams (Tam et al., 2005; Felton et al., 2013; Tam et al., 2017). However, more recent studies may support the strict need of attaining aggressive PK/PD targets of at least 100%*f*T_>4 x MIC_ for both maximizing clinical efficacy and suppressing resistance emergence in Gram-negative infections (Tam et al., 2005; Felton et al., 2013; Tam et al., 2017; Sumi et al., 2019; Al-Shaer et al., 2020; Alshaer et al., 2022; Chua et al., 2022; Alshaer et al., 2023; Gatti et al., 2023c). In the case of beta-lactam/beta-lactam inhibitor combinations (BL/BLIc), it has been recently proposed the so-called aggressive joint PK/PD target (Gatti et al., 2023b). Specifically, 100%*f*T_>4 x MIC_ of BL should be coupled with 100%*f*T of BLI above the target concentrations (C_T_) used by the EUCAST for testing *in vitro* the susceptibility of BL (Gatti et al., 2023b). In this setting, a recent study including 43 critically ill patients (of which 4.7% were OLT recipients) treated with continuous infusion (CI) piperacillin-tazobactam monotherapy for documented Gram-negative BSIs and/or VAP found that failure in attaining aggressive joint PK/PD target emerged as the only independent predictor of microbiological failure (OR 37.2; 95%CI 3.66–377.86; p=0.002) (Gatti et al., 2023c).

The attainment of aggressive PK/PD targets for beta-lactams may play an essential role in critically ill and/or in immunosuppressed patients affected by Gram-negative infections. In this regard, it should be noticed that failure in attaining aggressive PK/PD targets in critical patients was reported in approximately 80% of cases when beta-lactams are administered by intermittent infusion (De Waele et al., 2014). In this scenario, adopting altered dosing strategies based on CI administration and implementing a therapeutic drug monitoring (TDM)-guided approach may significantly increase the likelihood of attaining aggressive PK/PD targets in critically ill patients (Guilhaumou et al., 2019). Specifically, the proportion of critical patients failing in attaining aggressive PK/PD targets with CI beta-lactams ranged from 5% to 28% (Richter et al., 2019; Chiriac et al., 2021; Gatti et al., 2022; Hagel et al., 2022; Dräger et al., 2023). A previous retrospective study including 166 critically ill patients (6.6% OLT recipients) reported a proportion of failure in attaining aggressive PK/PD targets at first TDM assessment with CI piperacillin-tazobactam and meropenem of 4.9% and 13.5%, respectively (Gatti et al., 2022). A recent meta-analysis of eleven studies found that implementing a TDM-guided approach was associated with significantly higher attainment of optimal beta-lactams PK/PD targets compared to standard management (risk ratio [RR] 1.85; 95%CI 1.08–3.16) (Pai Mangalore et al., 2022).

Unfortunately, evidence on this topic in the specific setting of OLT recipients are limited. To this regard, we recently carried out a retrospective study among critically ill OLT recipients who during the early post-transplant period were treated with CI beta-lactams (i.e., piperacillin-tazobactam, meropenem, ceftazidime-avibactam, and meropenem-vaborbactam) and had treatment optimized in real-time by means of a TDM-guided expert clinical pharmacological advice (ECPA) program (Gatti et al., 2023b). Overall, 77 critical OLT recipients receiving 100 different beta-lactam treatment courses were included, and failure in attaining early aggressive PK/PD of beta-lactams was reported in 12% of cases. Notably, augmented renal clearance (ARC; OR 7.64; 95%CI 1.32–44.13) and MIC values above the EUCAST clinical breakpoint (OR 91.55; 95%CI 7.12–1177.12) emerged as independent predictors of failure in attaining early aggressive beta-lactam PK/PD targets (Gatti et al., 2023b).

### 3.2 Abdominal/biliary and lung penetration of beta-lactams

Attaining optimal PK/PD targets for beta-lactams at site of infection represents an essential requirement for maximizing clinical outcome in deep-seated infections occurring in OLT recipients (Pea and Viale, 2006). In this scenario, it is important to take into account not only the penetration rate of the different beta-lactams, but above all to assess whether the reported absolute concentrations at the site of infection may ensure the attainment of optimal PK/PD targets (Pea and Viale, 2009).

A summary of available evidence concerning biliary/abdominal and lung penetration of traditional and novel beta-lactams in OLT recipients are reported in **Table 1**.

**Table 1:** Summary of evidence concerning biliary/abdominal and lung penetration of traditional and novel beta-lactams in orthotopic liver transplant recipients.

Beta-lactam	Dosing regimen	Site of infection	Penetration rate	Absolute concentrations at site of infection	Attainment of optimal PK/PD target at site of infections
Ceftazidime-avibactam (Gatti et al., 2023a)	2.5 g q8h CI	Abdominal (peritoneal fluid)	0.78–1.14 (ceftazidime) 0.84–1.33 (avibactam)	Ceftazidime average C_ss_=71.2 mg/L* Avibactam average C_ss_=18.3 mg/L*	For MIC up to 8 mg/L
Cefotaxime (Buijk, 2004)	1 g q6h II 4 g/day CI	Bile	0.8–0.9	Average C_min_= 7.36 mg/L* Average C_ss_= 18.4 mg/L*	For MIC up to 4 mg/L
Ceftriaxone (Toth et al., 1991)	2 g/day	Bile	1.23	Average C_min_= 5.9 mg/L*	For MIC up to 1 mg/L
Cefoperazone-sulbactam (Muder et al., 2002)	3 g q12h II	Bile	17.3	Cefoperazone average C_min_= 43.6–411.8 mg/L* Sulbactam average C_min_= 2.0–5.4 mg/L*	For MIC up to 8 mg/L
Piperacillin (Dupon et al., 1993)	2.25 g q4h II	Bile	0.65–1.25	Average C_min_= 46.6 mg/L*	For MIC up to 8 mg/L

CI, continuous infusion; C_min_, trough concentration; C_ss_, steady-state concentration; II, intermittent infusion; MIC, minimum inhibitory concentrations; OLT, orthotopic liver transplant; PK/PD, pharmacokinetic/pharmacodynamic;

* After adjustment for a protein binding of 90% for ceftriaxone and cefoperazone; 38% for sulbactam; 20% for cefotaxime and piperacillin; 10% for ceftazidime; 7% for avibactam.

In regard to abdominal penetration of beta-lactams, evidence are limited to a single case report assessing penetration and joint PK/PD target attainment of ceftazidime-avibactam in peritoneal fluid in a critical OLT recipient affected by bacteremic complicated intrabdominal infection due to OXA-181-producing *Klebsiella pneumoniae* (Gatti et al., 2023a). A peritoneal fluid-to-plasma ratios of 0.78–1.14 and of 0.84–1.33 was found for ceftazidime and avibactam, respectively, allowing for the attainment of optimal joint PK/PD target throughout treatment course (Gatti et al., 2023a).

Good peritoneal exudate-to-plasma ratios were found for other beta-lactams (i.e., cefotaxime, ceftriaxone, ceftolozane-tazobactam, piperacillin-tazobactam, ertapenem, and meropenem) in critically ill non-OLT patients affected by complicated intrabdominal infections, ranging from 0.74 to 1.74 (Karjagin et al., 2008; Verdier et al., 2011; Leon et al., 2020; Yoshimura et al., 2023). Absolute concentrations in peritoneal fluid ensured the attainment of optimal PK/PD targets for all agents except for ertapenem (Karjagin et al., 2008; Verdier et al., 2011; Leon et al., 2020; Yoshimura et al., 2023).

In regard to biliary penetration of beta-lactams, few evidence are currently available in OLT recipients. Specifically, cefotaxime showed a bile-to-plasma ratio of 0.8–0.9 when administered by intermittent (1 g every 6 h) or CI (4 g/day) in 15 OLT recipients (Buijk, 2004). CI granted significant higher biliary cefotaxime average C_ss_ compared to C_min_ observed with intermittent infusion (18.4 mg/L vs. 7.36 mg/L), allowing the attainment of optimal PK/PD target for MIC up to the clinical breakpoint (Buijk, 2004).

In regard to ceftriaxone, average bile-to-plasma ratio of 1.23 was found after the administration of 2 g/day in seven OLT recipients (Toth et al., 1991). Biliary ceftriaxone average *f*C_min_ allowed to attain optimal PK/PD target for MIC up to the clinical breakpoint (Toth et al., 1991).

In regard to cefoperazone, average bile-to-plasma ratio of 17.3 was reported in nine OLT recipients treated with 3 g q12h of cefoperazone-sulbactam by intermittent infusion (Muder et al., 2002). Average biliary cefoperazone *f*C_min_ ensured the attainment of optimal PK/PD target for MIC up to the clinical breakpoint (Muder et al., 2002).

In regard to piperacillin, average bile-to-plasma ratio ranging from 0.65 to 1.25 was found in 10 OLT recipients treated with 2.25 g q4h of piperacillin-tazobactam by intermittent infusion (Dupon et al., 1993). Although biliary penetration of piperacillin was significantly lower than those reported in non-OLT recipients (0.65–1.25 vs. 5.3) (Thabit, 2020), absolute biliary piperacillin concentrations allowed to attain optimal PK/PD targets against *Enterobacterales* and *Pseudomonas aeruginosa* up to the clinical breakpoint of 8 mg/L (Dupon et al., 1993).

In regard to penetration of beta-lactams into epithelial lining fluid (ELF), no studies were conducted in OLT recipients. Overall, available evidence in critical ventilated non-OLT recipients reported a low-to-moderate ELF-to-plasma ratios for both traditional (i.e., ceftazidime, piperacillin-tazobactam, meropenem) and novel beta-lactams (i.e., ceftolozane-tazobactam, cefiderocol), ranging from 0.21 to 0.57 (Boselli et al., 2004a, Boselli et al., 2004b, 2008; Lodise et al., 2011; Felton et al., 2018; Benítez-Cano et al., 2020; Caro et al., 2020; Kawaguchi et al., 2022). Notably, absolute ELF concentrations of all these agents were inadequate for attaining optimal PK/PD targets against *Pseudomonas aeruginosa* with MIC values up to the clinical breakpoint.

### 3.3 Pharmacokinetic alterations of beta-lactams associated with pathophysiological alterations of OLT recipients

Beta-lactams exhibit common physicochemical and PK features, namely low molecular weight, hydrophilic properties, limited volume of distribution, low plasma protein binding, and predominant renal clearance (Veiga and Paiva, 2018). Consequently, the PK behavior of both traditional and novel beta-lactams may be significantly affected by different pathophysiological alterations commonly retrieved in critically ill patients (Roberts et al., 2012, 2014). Specifically, these pathophysiological alterations usually lead to remarkable variations in volume of distribution and clearance of beta-lactams, potentially resulting in failure in attaining optimal PK/PD targets (Abdulla et al., 2021). Furthermore, the remarkable proportion of hypoalbuminemia reported after transplantation may affect the PK behavior of beta-lactams exhibiting high protein binding (Ulldemolins et al., 2011; Roberts et al., 2012). Indeed, on the one hand, hypoalbuminaemia is likely to increase the volume of distribution and clearance of agents exhibiting high protein binding, potentially leading to lower antibiotic exposures that could affect the attainment of optimal PK/PD targets (Ulldemolins et al., 2011; Roberts et al., 2012, 2014). On the other hand, hypoalbuminemia (i.e., serum albumin levels < 3.0 g/dL) was significantly associated with a higher risk of acute kidney injury in 998 OLT recipients (Sang et al., 2015), thus potentially affecting PK behavior of beta-lactams independently from the degree of protein binding.

Currently, few evidence assessed the PK behavior of beta-lactams in OLT recipients (**Table 2**). An early study assessed the PK behavior and the PK/PD target attainment of ceftriaxone administered at 2 g/day in seven OLT recipients (Toth et al., 1991). Ceftriaxone showed in OLT recipients larger volume of distribution (Vd; 16.6 L vs. 10.1 L), longer elimination half-life (t_1/2_ 13.1 h vs. 5.8 h), and lower body total clearance (CL; 0.81 vs. 1.19 L/h) compared to historical healthy subjects (Toth et al., 1991). Total plasma C_min_ and optimal PK/PD target attainment of ceftriaxone were higher in OLT recipients than in historical healthy subjects (48 mg/L vs. 15 mg/L) (Toth et al., 1991). Furthermore, high variation in unbound ceftriaxone fraction was retrieved, ranging from 5% to 56% (Toth et al., 1991). Binding parameters of ceftriaxone (i.e., capacity constant and affinity constant) markedly differed from those calculating in historical healthy subjects, although no significant correlation with serum albumin levels was found (Toth et al., 1991).

**Table 2:** Summary of studies investigating PK behavior of beta-lactams in OLT recipients.

Beta-lactam	Number of OLT recipients	Dosing regimen	Mean plasma C_ss_ or C_min_	Attainment of optimal PK/PD target	PK parameters	Main PK alterations caused by OLT
Ceftriaxone (Toth et al., 1991)	7	2 g/day	48 mg/L vs. 15 mg/L (OLT recipients vs. healthy subjects)	100%*f*T_>4 x MIC_	Total body CL: 0.81 vs. 1.19 L/h (OLT recipients vs. healthy subjects) Vd: 16.6 vs. 10.1 L (OLT recipients vs. healthy subjects) t_1/2_: 13.1 vs. 5.8 h (OLT recipients vs. healthy subjects)	After OLT, increase in Vd and decrease in CL compared to healthy subjects
Cefotaxime (Buijk, 2004)	15	4 g/day CI 1 g q6h II	18 mg/L 2.2 mg/L	100%*f*T_>MIC_ 60%*f*T_>MIC_	Vd: 0.40 vs. 0.24 L/kg (OLT recipients vs. healthy subjects) t_1/2_: 3.6 vs. 1.2 h (OLT recipients vs. healthy subjects)	No significant correlation between blood loss and cefotaxime total body CL After OLT, increase in Vd, increase in t_1/2_, and decreased hepatic CL compared to healthy subjects
Cefoperazone-sulbactam (Muder et al., 2002)	9	3 g q12h II	NA	NA	Cefoperazone: CL: 0.53 vs. 0.23 mL/min/kg (intraoperative vs. postoperative period) Vd: 0.19 vs. 0.23 L/kg (intraoperative vs. postoperative period) t_1/2_: 4.4 vs. 17.4 h (intraoperative vs. postoperative period) Sulbactam: CL: 1.51 vs. 1.09 mL/min/kg (intraoperative vs. postoperative period) Vd: 0.26 vs. 0.34 L/kg (intraoperative vs. postoperative period) t_1/2_: 2.3 vs. 4.4 h (intraoperative vs. postoperative period)	PK parameters of cefoperazone and sulbactam were significantly altered in OLT recipients compared to healthy subjects, but similar to those observed in liver and renal impairment
Piperacillin (Dupon et al., 1993)	10	2.25g q4h II	46.5–55.2 mg/L	100%*f*T_>4 x MIC_	CL: 7.32 vs. 11.8 L/h (OLT recipients vs. healthy subjects) Vd: 23 vs 21 L (OLT recipients vs. healthy subjects) t_1/2_: 3.26–3.65 vs. 0.9 h (OLT recipients vs. healthy subjects)	Piperacillin plasma concentrations were quite similar to levels reported in healthy subjects despite large blood loss and fluid replacement
Meropenem (Morales Junior et al., 2023)	14	40 mg/kg q8h over 15 minutes or 3h	24.2–79.1 mg/L	100%*f*T_>4 x MIC_	CL: 0.39–0.97 vs. 6.5–7.2 mL/min/kg (OLT recipients vs. critically ill pediatric patients) Vd: 0.08–0.17 vs 0.2–0.4 L/kg (OLT recipients vs. critically ill pediatric patients) t_1/2_: 1.8–2.7 h (OLT recipients)	Large difference in total body CL and Vd were retrieved between pediatric OLT recipients and critical pediatric patients or healthy subjects

CI, continuous infusion; CL, clearance; C_min_, trough concentrations; C_ss_, steady-state concentrations; II, intermittent infusion; OLT, orthotopic liver transplant; MIC, minimum inhibitory concentration; PK/PD, pharmacokinetic/pharmacodynamic; t_1/2_, half-life; Vd, volume of distribution.

NA, not assessed.

A PK study evaluated the PK profile and the PK/PD target attainment of cefotaxime administered at the dose of 1g every 6 hours by intermittent infusion vs. that of 4 g/day by CI in 15 OLT recipients (Buijk, 2004). Cefotaxime showed in OLT recipients larger Vd (0.4 L/kg vs. 0.24 L/h) and longer elimination half-life (t_1/2_ 3.6 h vs. 1.2 h) compared to historical healthy subjects (Buijk, 2004). CI administration of cefotaxime granted much higher steady-state concentration in comparison with the C_min_ achieved by intermittent infusion (18 mg/L vs. 2.2 mg/L), thus granting better attainment of optimal PK/PD target (Buijk, 2004).

The PK behavior of cefoperazone-sulbactam administered at a dosage of 3 g every 12 hours by intermittent infusion was investigated in nine OLT recipients both in the intraoperative and in the post-operative period (Muder et al., 2002). In the post-operative period, both cefoperazone and sulbactam showed in OLT recipients a slightly higher Vd (0.23 L/kg vs. 0.19 L/kg for cefoperazone; 0.34 L/kg vs. 0.26 L/kg for sulbactam) and a longer elimination half-life (17.4 h vs. 4.4 h for cefoperazone; 4.4 h vs. 2.3 h for sulbactam) and lower total body CL (0.23 mL/min/kg vs. 0.53 mL/min/kg for cefoperazone; 1.09 mL/min/kg vs. 1.51 mL/min/kg for sulbactam) compared to historical healthy subjects (Muder et al., 2002). Overall, PK parameters of cefoperazone and sulbactam retrieved in OLT recipients were similar to those observed in patients having liver and/or renal impairment (Muder et al., 2002).

The PK behavior and the PK/PD target attainment of piperacillin administered at a dosage of 2.25 g every 4 hours by intermittent infusion were evaluated in ten OLT recipients (Dupon et al., 1993). No significant difference emerged between OLT recipients and healthy subjects in terms of Vd (23 L vs. 21 L) and total body CL (7.32 L/h vs. 11.8 L/h) (Dupon et al., 1993). Mean piperacillin C_min_ reported in OLT recipients (46.5–55.2 mg/L) allowed optimal PK/PD target attainment for MIC up to the clinical breakpoint (Dupon et al., 1993).

The PK behavior and the PK/PD target attainment of meropenem administered at a dosage of 40 mg/kg every 8 hours by intermittent (over 15 minutes) or extended (over 3 hours) infusion were assessed in 14 pediatric OLT recipients (Morales Junior et al., 2023). In pediatric OLT recipients both Vd (0.08–0.17 L/kg vs. 0.2–0.4 L/kg) and total body CL (0.39–0.97 mL/min/kg vs. 6.5–7.2 mL/min/kg) were significantly lower compared to critically ill pediatric patients or healthy subjects (Morales Junior et al., 2023). The high meropenem C_min_ observed pushed the authors to recommend the need of a TDM-guided strategy for minimizing the risk of overexposure in pediatric OLT recipients (Morales Junior et al., 2023).

Overall, these findings suggest that PK behavior of beta-lactams may be altered in OLT recipients compared to healthy subjects. The larger Vd, the longer elimination half-life, and the lower CL commonly observed with the different beta-lactams may result from vascular clamping and fluids redistribution associated with OLT procedure.

Other conditions potentially affecting PK/PD target attainment of beta-lactams administered as prophylaxis and/or treatment in the intra- or the early post-operative period are represented by blood losses and/or blood transfusions resuscitation (Swoboda, 1996). Currently, evidence on this topic are limited. A PK study including ten OLT recipients investigated the effects of blood resuscitation on exposure of ampicillin-sulbactam during transplantation (Lasko et al., 2022). OLT recipients received between 500 and 23,642 mL of total blood product. No statistically significant relationship was observed between blood resuscitation and ampicillin-sulbactam exposure (*R^2^ =* 0.00–0.26) (Lasko et al., 2022). Similarly, no significant correlation was reported between the magnitude of blood loss and the total body CL of cefotaxime (*R*=0.32) among 15 critical OLT recipients having a mean blood loss of 13 L during transplantation (Buijk, 2004). Furthermore, no significant differences in piperacillin exposure were reported among ten OLT recipients compared to healthy subjects despite a mean intraoperative replacement of 9.1 L and 3.2 L of blood products and crystalloids, respectively (Dupon et al., 1993).

Overall, these findings suggest that the PK behavior of beta-lactams in terms of total CL and exposure may be not significantly affected by massive blood and/or fluid resuscitation, resulting in limited impact on the attainment of optimal PK/PD targets.

Finally, the pharmacokinetics of beta-lactams may be affected also by systems providing renal replacement therapy, removing endotoxins, and adsorbing cytokines even simultaneously, namely CytoSorb hemoadsorption device, oXiris hemofilter, or the Molecular Adsorbent Recirculating System (MARS), which are important supportive tools in patients with severe liver failure or OLT recipients (Isoniemi et al., 2005; Li et al., 2022; Bottari et al., 2023; Popescu et al., 2023). Although no specific study was conducted in the setting of OLT recipients, interesting data may be retrieved by preclinical studies and by clinical studies conducted in other settings of critically ill patients. In this regard, an experimental animal model assessed the PK of 17 different antimicrobials during hemoadsorption with CytoSorb. The impact of adsorption was defined as mild, moderate, or strong when the baseline drug CL was increased by >25%, >100%, and >400%, respectively (Schneider et al., 2021). The impact on all of the evaluated beta-lactams was negligible, specifically +19.4% for piperacillin, +6.3% for meropenem, +5.2% for ceftriaxone and +1.2% for cefepime (Schneider et al., 2021). A PK model assessing whether meropenem CL was affected by CytoSorb treatment in 25 critically ill patients found a negligible impact with an increase < 3.7% (Liebchen et al., 2021). Similarly, a prospective observational study carried out in ten critically ill patients undergoing hemoadsorption treatment with CytoSorb reported that drug CL was increased by 43% for ceftazidime and decreased by 57% for meropenem (Bottari et al., 2023). In regard to the use of oXiris high-adsorbent membrane, a prospective population PK study was conducted among 12 critically ill patients treated with meropenem at a dosage of 1000 mg every 8 hours (Padullés Zamora et al., 2019). The findings showed that under this condition the meropenem dose needed for attaining optimal PK/PD target of 100%*f*T_>4xMIC_ against *Pseudomonas aeruginosa* should be of 3000 mg/day by CI or of 2000 mg every 8 hours by extended infusion (Padullés Zamora et al., 2019). In regard to MARS, one case reported the impact of this method coupled with continuous venovenous hemodialysis (CVVHD) on the CL of piperacillin-tazobactam administered at a dosage of 4.5 g every 6h over 3h-infusion. An increase of 2.9-fold in the piperacillin elimination rate constant and a 3.7-fold reduction in t_1/2_ during MARS therapy compared to CVVHD alone was documented (Ruggero et al., 2013).

Overall, these findings suggest that beta-lactam total CL may be increased during the application of oXiris high-adsorbent membrane or MARS, whereas a negligible impact could be expected for CytoSorb treatment in OLT recipients.

## 4 Conclusions and future perspectives

Overall, studies concerning the optimization of beta-lactam treatments according to the “antimicrobial puzzle” principles and the implementation of a real-time TDM-guided approach in critical OLT recipients are currently limited. However, some relevant concepts may be inferred according to available evidence in critical OLT and non-OLT patients.

Firstly, attaining aggressive beta-lactams PK/PD targets (i.e., at least a 100%*f*T_>4xMIC_) may be strongly recommended in the critically ill, particularly in immunosuppressed patients. In this regard, a recent meta-analysis including 21 observational studies with a total of 4,833 patients with a vast proportion of OLT recipients (up to 100%) found that attaining aggressive PK/PD targets was significantly associated with higher clinical cure rate (OR=1.69; 95% CI 1.15–2.49) and lower risk of beta-lactam resistance development (OR=0.06; 95% CI 0.01–0.29), whereas failure in attaining these targets was significantly associated with higher risk of microbiological failure (OR=26.08; 95% CI 8.72–77.95) (Gatti et al., 2024). Consequently, maximizing the attainment of aggressive beta-lactam PK/PD targets may represent one of the main goals in the treatment of critical OLT recipients with suspected and/or documented sepsis.

Secondly, only few evidence currently assessed abdominal and biliary penetration of beta-lactams in OLT recipients, whereas no studies or case reports investigated beta-lactam penetration in ELF in this scenario. Although it could be expected that no remarkable difference could be retrieved in terms of abdominal and lung penetration for both traditional and novel beta-lactams in critical OLT recipients with respect to critical non-OLT patients, the available evidence assessing beta-lactam penetration rate and PK/PD target attainment at infection site should be applied cautiously in OLT recipients, considering that OLT-associated pathophysiological alterations potentially affecting the penetration of beta-lactams in deep-seated infections could not be ruled out. Consequently, further evidence assessing the penetration rate and PK/PD target attainment of beta-lactams in the specific scenario of critical OLT recipients are strongly warranted. Although biliary penetration and absolute concentrations were consistent with those retrieved in non-OLT patients for the different beta-lactams (Thabit, 2020), piperacillin showed a significant lower penetration and absolute concentrations in first bile secretion after OLT compared to non-OLT patients (Dupon et al., 1993; Thabit, 2020). In this scenario, beta-lactam concentrations in the first bile secretion after transplant when liver function is not yet fully established may be significantly lower compared to those retrieved in non-OLT recipients with normal liver function (Dupon et al., 1993), potentially affecting the attainment of optimal biliary PK/PD targets. Consequently, evidence investigating beta-lactam penetration in bile in non-OLT patients should be applied cautiously in OLT recipients (Thabit, 2020), and the assessment of liver function, bile quality, and time elapsed from grafting should be mandatory for estimating possible biliary penetration of a specific agent.

Thirdly, specific OLT-associated pathophysiological alterations may potentially affect the attainment of beta-lactams aggressive PK/PD targets in critical OLT recipients. Unfortunately, studies assessing the impact on beta-lactam exposure of these specific alterations are limited or completely lacking. In regard to massive blood losses and transfusions during liver transplantation, few studies reported no significant impact on beta-lactam exposure and/or PK/PD target attainment (Dupon et al., 1993; Buijk, 2004; Lasko et al., 2022). In this scenario, it could be expected that the decrease in total CL may counteract for the increase in Vd after massive blood resuscitation, thus justifying the lack of impact on beta-lactams exposure in critical OLT recipients with massive blood losses during intervention. Indeed, it is noteworthy that vascular clamping of all hepatic vessels, portal vein, and inferior vena cava above renal veins during intervention may temporary induce hepatic and renal failure, potentially resulting in decrease beta-lactam CL despite the massive blood losses and transfusions (Arnow et al., 1992). Further evidence assessing the impact of the different hemoadsorption and/or hemofilter devices on the PK behavior of antimicrobials are also strongly warranted. Few preclinical and clinical studies suggested a negligible impact of the CytoSorb hemoadsorption device on the CL of hydrophilic agents, including beta-lactams. Conversely, the implementation of oXiris high-adsorbent membrane or MARS may potentially affect the PK behavior of beta-lactams, with consequent need for higher dosing according to an increased CL.

Fourthly, adopting a real-time TDM-guided approach could be helpful with the aim of maximizing the attainment of beta-lactam aggressive PK/PD targets, promptly identifying (i.e., in the first 24–48 hours) OLT recipients at high-risk for failure in attaining optimal PK/PD targets, and minimizing the risk of toxicity due to overexposure. Notably, up to 12% of critical OLT recipients treated with beta-lactams failed in attaining early aggressive PK/PD targets despite the administration by CI (Gatti et al., 2023b). In this scenario, implementing tools or risk score able to promptly identify cases at high-risk for failure in attaining aggressive PK/PD targets could be helpful for selecting appropriate beta-lactam dosing before the first TDM assessment (Abdulla et al., 2021; Gatti et al., 2024). In this regard, a timely and successful real-time TDM-based ECPA program should include a well-defined dedicated laboratory pathway (Gatti et al., 2022; Gatti and Pea, 2023b). Bioanalytical experts should be directly involved in this project for properly addressing lab issues. Specific analytical methods for each of the different beta-lactams should be developed and validated by means of liquid chromatography-mass spectrometry or high performance liquid chromatography for accurately measuring drug concentrations in plasma/serum. Planning multiple daily TDM sessions with short turnaround times (TATs) is key element for allowing the MD Clinical Pharmacologist to provide prompt advices to clinicians on how adjusting beta-lactam dosing regimens in critical OLT recipients.

**Figure 2:**
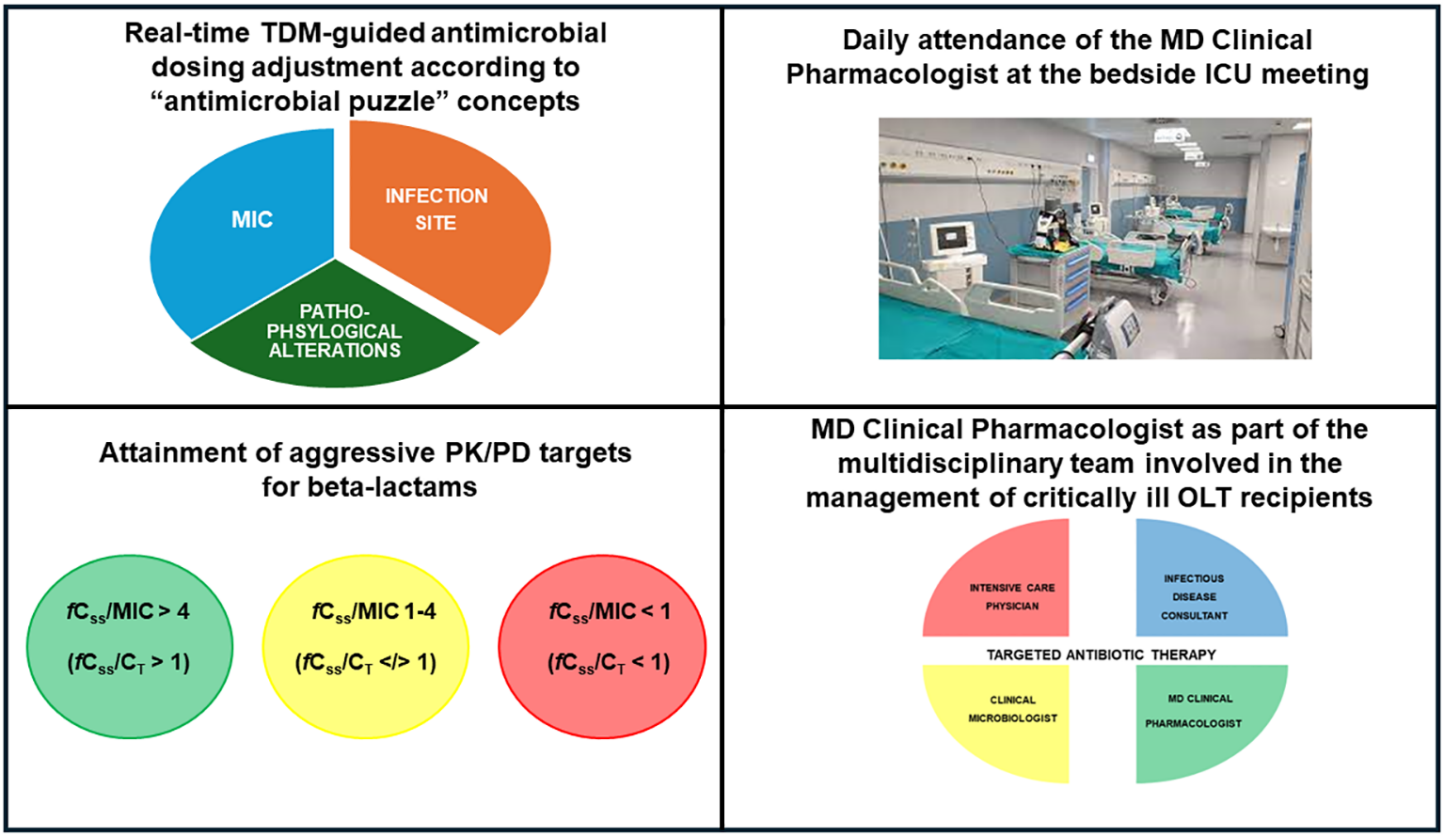
Main features of real-time TDM-guided ECPA program for optimizing PK/PD target attainment of beta-lactams and role of the MD clinical pharmacologist in the multidisciplinary team involved in the management of critical OLT recipients. *f*C_ss_, free steady-state concentrations; C_T_, target concentration; ICU, intensive care unit; MIC, minimum inhibitory concentration; OLT, orthotopic liver transplant; PK/PD, pharmacokinetic/pharmacodynamic; TDM, therapeutic drug monitoring.

Finally, implementing a coordinated and synchronized multidisciplinary team including the intensive care physician, the surgeon, the infectious disease consultant, the hepatologists, the clinical microbiologist, and the MD clinical pharmacologist could play a major role in the management of critical OLT recipients affected by suspected or documented sepsis (Patel and Huprikar, 2012; Lucey et al., 2023). Previous evidence supported the role of a multidisciplinary management team in improving clinical outcome in different settings involving critically ill patients (Viale et al., 2017; Gatti et al., 2019; Rinaldi et al., 2024). To this regard, a recent pre-post quasi-experimental study assessed the role of a multidisciplinary team composed by the intensivist, the infectious disease consultant, the clinical pharmacologist and the microbiologist on the outcome of 135 critical patients (10.3% OLT recipients) having documented Gram-negative BSIs (Rinaldi et al., 2024). In the post-intervention phase, daily evaluation by the multidisciplinary team was linked to significant decreases of either microbiological failure (10.3% vs. 29.9%; p=0.005) or 30-day new-onset colonization by multidrug-resistant organisms (8.3% vs. 36.6%; p<0.001) (Rinaldi et al., 2024). In the scenario of critical OLT recipients, the MD clinical pharmacologist should act in a synchronized way with the other members of the multidisciplinary team, with the aim of ensuring both the optimization of beta-lactam dosing according to “antimicrobial puzzle” concepts, including the careful assessment of specific OLT-associated pathophysiological alterations, and the promptly attainment of aggressive PK/PD targets by adopting a real-time TDM-guided ECPA program (**Figure 2**). It is noteworthy that the daily attendance of the MD Clinical Pharmacologist at the multidisciplinary bedside ICU meeting may represent an added value, especially for ensuring a prompt management of sudden variations in organ function which usually occur in critical OLT recipients and consequently affect beta-lactam exposure and PK/PD target attainment.

In conclusion, several research gaps still exist in assessing the PK behavior of beta-lactams in critical OLT recipients. The impact of specific OLT-associated pathophysiological alterations on the attainment of optimal PK/PD targets may represent an important field in which further studies are warranted. Assessing the relationship between aggressive beta-lactam PK/PD target attainment and clinical outcome in critical OLT recipients will represent a major challenge in the next future.

## Author contributions

MG: Writing – original draft, Methodology, Data curation, Conceptualization. FP: Writing – review & editing, Conceptualization.
